# Supplementary material for: Interactions of the Insect-Specific Palm Creek Virus with Zika and Chikungunya Viruses in Aedes Mosquitoes
Source: Microorganisms. 2021 Aug 3;9(8):1652. doi: 10.3390/microorganisms9081652 (PMC8402152; doi:10.3390/microorganisms9081652)
Supplement: Supplementary file 1 [file microorganisms-09-01652-s001.zip › Figure S1.pdf]

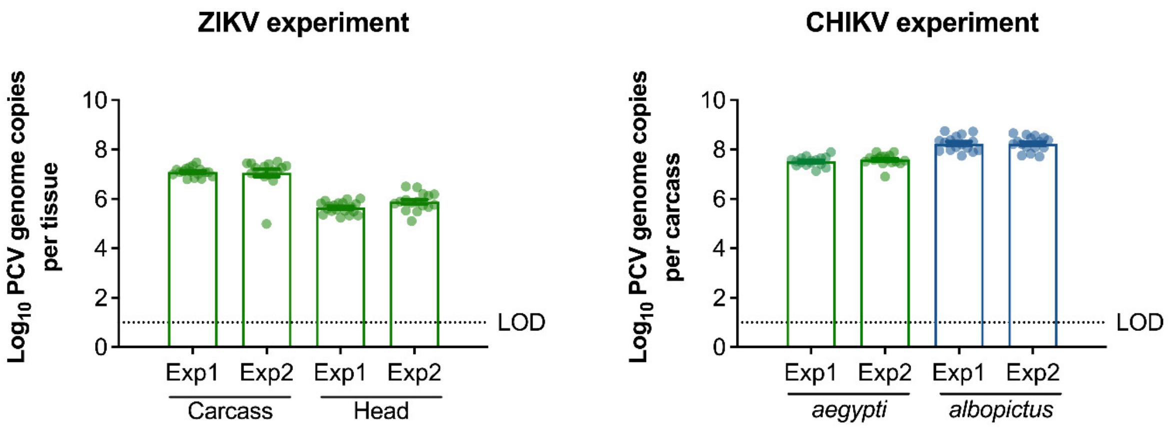

Figure S1. Palm Creek virus titres in intrathoracically-injected *Aedes aegypti* and *Ae. albopictus* mosquitoes used in Zika virus (ZIKV) and chikungunya virus (CHIKV) inhibition experiments as quantified by qRT-PCR. Each data point denotes an individual mosquito pooled across two experimental replicates. Bars depicting means, standard errors, limit of detection (LOD) are shown.
